# Traité Pratique des Maladies de l'Uterus et de ses Annexes, &c. Practical Treatise on Diseases of the Uterus, &c.

**Published:** 1833-04-01

**Authors:** 


TRAITÉ PRATIQUE DES MALADIES DE L'UTERUS ET DE SES ANNEXES, FONDÉ SUR UN GRAND NOMBRE D'OBSERVATIONS CLINIQUES; accompagné d'un Atlas de 41 Planches in folio, gravées et coloriées. Par Madame *Veuve Boivin*, Docteur en Médecine, Sage Femme surveillante en chef de la Maison Royale de Santé, &c. &c. et par *A. Dugès*, Professeur à la Faculté de Médecine de Montpellier, &c. &c. Tome Premier. 8vo. pp. 399. Baillière, Paris et Londres, 1833.

BOTH ourselves and the public will be much indebted to M. Baillière for bringing this work before us. Our readers must not be alarmed at perceiving that one of its authors is a lady. Madame Boivin has attained a deservedly high reputation, and her experience in female diseases has been very great. We have, on former occasions, directed attention to insulated papers of this lady's, and the work before us enhances our opinion of her merit.

We do not think it necessary to enter on a systematic analysis of the work. We shall select some particular chapters for notice, and, first, of anteversion of the uterus.

### Anteversion of the Uterus.

This displacement, though extremely common, is frequently neglected, and unknown both to writers and practitioners. The uterus is naturally inclined forwards, and when its fundus or anterior part is enlarged, this disposition is of course increased, the utero-iliac peritoneal ligaments are stretched, and, in certain positions of the female, the fundus is carried forwards on the bladder, whilst the os tincæ is borne backwards and upwards on the rectum.

Although increase of bulk is the ordinary cause of anteversion, it rarely occurs in pregnancy, because here the enlargement is general, and the organ is carried out of the pelvis. The only example of anteversion during pregnancy, known to our authors, was communicated by Chopart to Baudelocque ; they have themselves seen one other instance. The fundus of the uterus was inclined forwards, and was lower than the neck, so that reduction appeared impossible ; however, as the volume of the uterus increased, the displacement passed away.

Our authors have seen, in many instances, the fundus of the uterus inclined forwards, to a degree intermediate between obliquity and anteversion, and they think it may be a not uncommon cause of difficult delivery, especially if the placenta is attached anteriorly. It seems that the uterus may be anteverted, independently of any enlargement of the organ. This may take place in consequence of repeated efforts, as, for example, those of vomiting, of straining at stool, repeated attempts at coitus, &c. Morbid adhesions, from inflammation of the uterus and peritoneum, may, by their retraction, produce the effect in question. In a case communicated by Madame Legrand to M. Ameline, adhesions of the os tincæ to the posterior wall of the vagina appeared to be the cause of an incurable anteversion.

Pain is most commonly experienced in the lumbar region, and extends thence to the epigastric. A feeling of weight in the region of the rectum and of the bladder is felt when the woman rises, walks, or even when sitting. The fundus of the uterus pressing on the bladder occasions a frequent desire to make water, or may even go so far as to give rise to partial retention of urine, whilst the os tincæ, carried against the rectum, impedes the course of the fæcal matter. These symptoms are not observed when the patient is reclined upon her back, unless the uterus be retained in its unnatural position by adhesions. The resemblance of the symptoms, in many respects, to those of calculus in the bladder, have led to a serious mistake. M. Levret performed the operation of lithotomy under these circumstances, and the patient's death gave him an opportunity of discovering that the case was one of anteversion only. Passing over the obvious diagnostic marks of the two affections, we may simply observe, that examination per vaginam is the most certain means of discriminating the case. If the patient be examined in the upright position, or seated at the edge of a chair, the portion of the uterus presented to the finger of the operator is its anterior face. Sometimes the vagina is partially obstructed by the fundus of the uterus, which has fallen forwards, or may, perhaps, be inclined to one side. The os tincæ is always much backwards, often to be looked for in the concavity of the sacrum. In general it is depressed, as is the whole of the uterus—occasionally so elevated, that the finger can scarcely touch it.

If the fore-finger be bent into the form of a hook, and the os uteri drawn forwards by its means, and then, if the finger be straightened and the fundus of the uterus pushed backwards, it is easily restored to its natural position, though it quickly returns to its unnatural one. If, however, there be adhesions, even this temporary reduction is unattainable.

The uterus, in these cases, is in general of greater volume, and possessed of more sensibility than natural. This may be either the consequence or the cause of the anteversion. Many believe that chronic inflammation is always the consequence; but our authors have seen several instances of acute metritis, after labour, subsiding into chronic, and producing anteversion, which could not be cured till it was removed.

It is in consequence of these observations, and of their belief in the frequency of deromé metritis as a cause of anteversion, that our authors are rather averse to the employment of mechanical means of treatment, such as pessaries. They have recourse to leeches to the anus and vulva, baths, enemata, rarely vaginal injections, narcotics, and the horizontal position on the back, with the pelvis a little raised. When there is little or no tenderness, and inconsiderable swelling, our authors agree to the use of mechanical means.

The pessary commonly employed is of the ball-and-socket kind (en bilboquet), the hollow being deep, to receive the neck of the uterus. This organ having been returned to its normal position, the patient lying on her back, the instrument is to be carefully introduced and adjusted. A plug-pessary (en boudon), particularly of the form of those denominated elythroides by M. Cloquet, may be sufficient in slight cases. But in some, more rare, a small sponge introduced into the vagina, behind a very prominent os tincæ, has retained the uterus in its position, when not very much deranged.

Many cases of anteversion are related by our authors. We need not particularize any, as we are anxious to pass to other portions of the volume.

Passing over chap. iv., on Retroversion of the Uterus, we stop at the fifth, which treats of hernia of the organ. And, first, of *ventral hernia*.

This occurs through an accidental opening in the abdominal muscles and aponeuroses. It has only been known to occur in the state of pregnancy, and has been confounded with an extreme anterior or latero-anterior obliquity of the uterus. We will briefly mention the three facts collected and related by our authors.

*Case* 1. A woman, who had been three days in labour, suddenly experienced violent pain in the belly. She was examined by J. L. Petit, who found a ventral hernia, extending from the umbilicus to the os pubis, and another from the umbilicus to the xiphoid cartilage. The inferior one was so large, that the recti muscles were separated from each other nine or ten inches. M. Petit was informed that the tumour had commenced a long time previously, that it had increased in size at each pregnancy, that the increase had been most rapid during the last six months ; but that it had only attained its actual size within the last three days.

*Case* 2. A woman who had had an enormous abscess, followed by cicatrices in the abdominal parietes, becoming pregnant, observed the cicatrix gradually yield, and the uterus descended at last as low as the knees. At the time of labour the tumour was supported, and delivery took place without difficulty. This case is related by Ruysch.

*Case* 3. A woman, aged 49, observed in her fifth pregnancy that a tumour, which had existed for some years towards the inguinal region, gradually enlarged, and it soon became evident that it contained the uterus and a living fœtus. Professor Saxtorph allowed labour to proceed ; but in endeavouring to extract the placenta, he again had occasion to remark the unnatural position of the uterus. The patient did well, but the uterus continued to protrude through the abdominal parietes, and that by a lacerated opening.—(*Bibliot. Med. t.* 67.)

*Crural Hernia of the Uterus.* In a case related by Samert, and in another by Doringius, the uterus formed a tumour situated towards the groin, but whether it protruded through the crural ring or not, it is difficult to say. In both cases, the uterus, being irreducible, was opened, as in the Cæsarian operation. Both patients died, but the infants lived. A case, however, has been recorded by M. Lallemand, in which the uterine hernia was clearly crural. The patient was eighty-two years of age at the time of her death. and the tumour had existed since the age of forty. It occupied the fold of the right groin, its form was pyramidal, its length five inches, its breadth four. The uterus had passed out behind the ligament of Fallopius, and was accompanied by the ovaries, Fallopian tubes, and a part of the vagina. The cause appeared to have been an omental hernia, which had taken place a few days after a natural accouchement. In fact, the reporters believed that the uterine protrusion had occurred only eight years before the patient's death.

*Inguinal Hernia of the Uterus.* If the possibility of the existence of such a hernia, during the condition of pregnancy be doubted, it is certain that it may occur when the uterus is unimpregnated. In a woman, aged 71, at the Salpetrière, the uterus, with the right ovary and a portion of the vagina, was enclosed in a thick herniary sac, which had passed through the inguinal ring on the right side. The form of the tumour was that of a pear, and it was four or five fingers' breadths in length. A similar case has been published by Chopart. The patient was fifty years of age. Nearly the whole uterus, with the left Fallopian tube and ovary, protruded through the inguinal ring on the left side, and was contained in a very large hernial sac. It was unusually small, elongated, contracted in the narrowest part of the ring, pale, and had attached to its fundus some membranous layers, which appeared to result from the detachment of the omentum, which had formerly adhered.

This species of hernia may be congenital, an instance of which has been given by our authors, in their general considerations on the genital organs. The subject was one of doubtful sex, and the hernia existed on the right side. The greater number of uterine herniæ have taken place on the right side, as, indeed, is the case with omental and intestinal protrusions.

Our authors conclude the chapter with a brief summary of the general characters of uterine herniæ. If the uterus be impregnated, the disease may be recognized by the volume of the tumour, its gradual increase, an obscure fluctuation, the motions of the fœtus, and the sounds of its heart as distinguished by the stethoscope. If, on the contrary, the uterus be unimpregnated, the hardness of the tumour, its rounded form, the thickness of the pedicle ; and, on the other hand, the tension and obliquity of the vagina, the elevation and disappearance of the os tincæ, are the diagnostic marks.

With respect to treatment, the reduction of the herniary uterus ought to be attempted, and, if effected, maintained in all cases, but more especially at the commencement of pregnancy. If irreducible, and the woman is past the age of child-bearing, a bandage or a suspensory will be sufficient ; if her age be less advanced, we may attempt gradual compression, according to the method of Petit in cases of adherent intestinal hernia. If pregnancy has taken place, we may attempt, as in case 3 of ventral hernia, to assist Nature, by raising and supporting the tumour, and making its axis the most convenient possible for delivery. If, however, we cannot effect this, the Cæsarian operation should be resorted to, dangerous as undoubtedly it is. If the hernia is in a state of strangulation, we must operate to relieve the stricture, as in other cases of hernia.

### UNNATURAL FIXEDNESS OF THE UTERUS.

This is the subject of chapter vi. In the former chapters, our authors have portrayed inconveniences arising from the mobility of the uterus ; in this they have to shew, from facts, what contrary inconveniences may arise from the organ being unnaturally restrained.

In youth both metritis and peritonitis are unfrequent. Chronic inflammation of the peritoneum, however, in early life, particularly when it affects the pelvic portion of it, may give rise to adhesions more or less firm and strict between the surface of the uterus and the neighbouring parts. The uterine appendages may adhere to that viscus, or to the pelvic parietes, and form ligaments, the inconveniences of which may be first experienced at a very distant period from that of their formation.

In mature age metritis and metro-peritonitis are much more frequent, whether from dysmenorrhœa, or difficult parturition. From these morbid adhesions may result. In a work on one of the most frequent causes of abortion, published by Madame Boivin, these adhesions were attentively considered, and their importance properly appreciated. Barrenness may result from the occlusion of the adherent fallopian tubes ; but conception in these cases may be worse than even barrenness, the traction exercised by the growing uterus lighting up perhaps fresh peritonitis. Such adhesions may give rise to pains and dragging sensations in the pelvis, a sense of lassitude in the lower extremities, formation of abscess in the neighbourhood of the vagina or rectum, and sometimes death. Commonly these untoward symptoms are preceded by abortion, occurring between the third and fifth month, and preceded and followed by hæmorrhage and all the signs of metritis.

These physical obstacles to the ascent of the uterus in pregnancy may be predicted from examination of the vagina and a careful consideration of the previous circumstances of the case. If the finger be introduced into the vagina, the uterus is generally felt depressed, or inclined to one side or other, and firmly fixed in that position. One of the round ligaments shortened, enlarged, may bind down the uterus. In this case the uterus rises to a greater height than in the other instances, but it is inclined, disturbed, and, as in a case related by Madame Boivin, it may give rise to premature labour: for example, at seven months.

We have said that it is necessary to inquire into the previous history of the case—to ascertain whether there have been symptoms of metritis, peritonitis, dysmenorrhœa, abortion, laborious parturition, wounds or abscess in the pelvic region. Our authors have remarked that unnatural fixedness of the uterus is most common in women of a lymphatic temperament, subject to constipation, and irregularities of the digestive organs.

Can adhesions, supposing them proved to exist, be removed? It is not likely that they can be so, but a proper antiphlogistic treatment may prevent their formation, and when dependent on persisting chronic inflammation, mercury, in removing that inflammation, has appeared to our authors to exercise an influence on the adhesions themselves.

Adhesions however may occur when the uterus is in a state of enlargement, and contains the developed ovum. The omentum may, under these circumstances, be firmly attached to the uterus while residing in the abdomen, and whilst it continues there no unpleasant symptom is observed. But when, after parturition, the uterus is rapidly re-descending into the pelvis, the omentum previously consolidated by inflammation is violently stretched, if too firm and resisting to give way, Peritonitis is re-induced, or, the uterus being unable to descend into the pelvis and contract, the orifices of the uterine sinuses are not adequately closed, and alarming or fatal hæmorrhage ensues. Baudelocque saw an instance of death in the early periods of pregnancy. The patient, in whom the omentum, rolled into the form of a cord, adhered to the anterior part and right side of the uterus, so that the stomach and arch of the colon were singularly dragged upon, had vomiting, diarrhœa, and syncope, previous to dissolution. In other cases related and represented by Ruysch and mentioned by Morgagni, painful dragging sensations and disturbance of the health were less serious consequences of such adhesions.

Five cases are related by our authors. We shall merely mention the particulars of two.

*Case.* Madame Delama, æt. 32, of extremely lymphatic temperament, was brought to the Maison de Santé, in a state of extreme prostration. She had menstruated at the age of twelve years, but very irregularly, and had been habitually subject to fluor albus and obstinate constipation. She married at the age of 25, and immediately contracted syphilis. At the age of 30 she became pregnant, and had a fortunate accouchement, followed by a return of the fluor albus. She again became pregnant, and at three months miscarried, after which there was uterine hæmorrhage for four days. That hæmorrhage was arrested on the evening before her admission into the Maison de Santé. On examining the uterus the os tincæ was found more enlarged and more firm than usual—the uterus was quite immoveable—the abdomen was somewhat blown up, and slight fluctuation was perceptible—and the left lower extremity was infiltrated throughout its whole extent. We need not particularize the treatment. The patient died on the 20th day after the miscarriage.

On dissection, a great quantity of yellow serous fluid flowed from the abdomen, when that cavity was opened. There were tuberculous granulations in the sub-peritoneal tissue of the iliac colon and of the rectum, both of which adhered on the one side to the uterus, and on the other to the sacrum. A vast abscess occupied the recto-vaginal duplicature of the peritoneum. The ovaria and fallopian tubes formed on the surface of the uterus, below the adhesions already mentioned, an inextricable mass infiltrated with pus.

*Case.* This patient was of a good constitution. At the age of seven years the menses appeared, and returned twice at intervals of a month. They then disappeared and did not return till the age of 10, after which they occurred regularly. At the age of 13 there was a profuse hæmorrhage from the uterus; at the age of 14½ this person married; at 16 she was a mother. At the age of 20 she broke her left femur, and ever afterwards was subject to pain in the left groin. About the age of 23 she had three abortions in fourteen months, one at four months, another at two months, the third at five months. From that time the menstruation was irregular, and she did not again become pregnant. In December 1819, whilst having connexion with her husband, a profuse uterine hæmorrhage occurred, with severe pains in the right iliac region. These symptoms were relieved by local bleeding, emollients, and narcotics.

In February, 1820, she had a relapse. On examination the uterus was found very low, resting on the perinæum, its neck tender and swollen was surrounded by the upper part of the vagina, and the uterus could scarcely be at all pushed up, whilst the attempt produced pains in the groin and hip. Constipation was extremely obstinate. Emollients, narcotics, &c. having been employed without permanent benefit, mercurial inunction was employed for a month. It did not produce salivation, but the pain and other unpleasant symptoms were completely relieved, and the reporters state that so much was the patient altered for the better, that she could scarcely be recognized. They forget to state, however, whether the immobility of the uterus was diminished.

The third section of this interesting volume is dedicated to the consideration of Alterations of the Form and Volume of the Uterus. It includes three chapters. The subject of the first is, 1, Partial Flexion of the Cervix Uteri; 2, Reversion (rebroussement) of the Os Tincæ; 3, Adhesions, Obliterations; 4, Elongation of the Cervix; 5, Atrophy, Hypertrophy. To this chapter then we will first advert.

*Partial Flexion of the Cervix Uteri.* It is well known that in the commencement of labour, the os uteri very little, if at all, dilated, is directed towards the sacrum, and sometimes even felt with difficulty. The anterior wall of the cervix then answers to the area of the vagina, and on it the weight of the fœtus and the brunt of the uterine contractions bear, and it also dilates, and yields the most. It is not to be wondered at that it sometimes becomes relaxed, and contracts with less facility than the opposed part. M. Dubois makes mention of a curious case.

A woman whose pregnancy had been accompanied by anasarca, &c. and who remained in the Infirmary of the Hospice de Maternité, at Paris, presented, on examination, in front of the neck of the uterus, a large tumour. The tumour was soft, large, rounded, and communicated to M. Dubois the idea of its resulting from an anteversion of the uterus, had not the fundus of this organ been distinctly felt in the abdomen. The bladder was empty, and the tumour without fluctuation. M. Dubois could only imagine it a fibrous tumour. The dropsy increased, and at the end of some days the patient died. On opening the body the tumour could not be discovered. The uterus was healthy, but softened, and the anterior wall preserved still a great tendency to be folded in front and below; and M. Dubois entertains no doubt that it was such a fold that formed the tumour.

*Reversion of the Os Tincæ.* In the early stage of cancer uteri it occasionally happens that the internal surface of the cervix becoming gorged and swollen, forces open, and everts the opposed surfaces of the orifice. We notice this reversion or eversion of the os tincæ, merely for the purpose of introducing a case, possessed of physiological interest.

*Case.* On the 8th November, 1832, a young woman was brought to the Maison de Santé in a dying state. She was said to have laboured under an inflammatory fever. On the day after her admission she died. On opening the body no other cause of death than a general engorgement of the vascular system was discovered.

The menstrual epoch had arrived during the latter days of the patient's life. The uterus was gorged with blood and of a violet colour, the ovarian vessels, especially the veins, were extremely dilated, the inner surface of the uterus of a bright red colour and smeared with colourless mucus. The tissue of the organ was soft, and the neck contained some *ova Nabothi*, of the size of a millet seed. The os tincæ was in a state of reversion, so that the surface generally concealed within the uterine cavity looked outwards. The young woman presented in the external organs all the appearances of chastity, but the ovaries were studded with cicatriculæ, and the right presented an erosion three or four lines in extent.

This reversion of the os uteri might possibly be mistaken, on a superficial examination, for ulceration. Pregnancy would probably remove it.

*Adhesions and Obliterations of the Os Tincæ.* Numerous as are the agencies which might seem calculated to produce ulcerations and adhesions of the lips of the os tincæ, such adhesions are in point of fact unfrequent. Our authors have found in elderly women the os tincæ annulated, wasted, destroyed, sometimes leaving in its place some bridles, and a funnel-like orifice, sometimes barely permitting the passage of a thread through its aperture. A case has been related by M. Ameline, in which such adhesion in earlier life produced retention of the menses in the uterus, and serious consequences. In other cases the same lesion would appear to have been productive of anteversion. Sometimes these adhesions oppose an obstacle to delivery, but not to impregnation, a circumstance which may result from the adhesions not having been so perfect as to have prevented the fecundating influence, or that they were not completed or not commenced until after impregnation. For cases of this kind, and of various degrees of contraction or obliteration of the os tincæ our authors refer to various publications.

*Elongation of the Cervix.* In certain cases of prolapsus, our authors have seen the whole lengthened. But elongation of the cervix only must not be mistaken for prolapsus. Sometimes the entire cervix is thus lengthened, and the reflexion of the vagina is thrown to a considerable distance from the free end of the cervix. Sometimes only one of the lips of the os tincæ acquires this elongation, and it may proceed to such an extent as to cause the lip to protrude from without the vulva. Professor Lallement has seen some cases of this kind in females much advanced in life, and before him Leroux of Dijon, appears to have witnessed something similar, but in pregnancy only. The latter gentleman observes, that sometimes one lip only, sometimes the entire cervix protrudes. He has seen it passing out of the vulva, and looking like the neck of a bottle, with its thickened border. On introducing the finger into the opening of the cervix, he could pass it as high as the internal orifice, which was closed by the membranes. When labour-pains commenced the cervix diminished and became gradually obliterated, in proportion as the internal orifice dilated.

In a case of elongation analogous to that described by Leroux, but in which the uterus was not gravid, the presence of the orifice at the summit of the elongation did not prevent a surgeon from believing in the existence of a polypus, and applying a ligature. The patient died of peritonitis. In a case related by Buisson, a pessary was applied. Bichat observed this elongation in the bodies of two or three individuals.

The second chapter of this section is devoted to the consideration of—

### INCURVATIONS OR FLEXIONS OF THE UTERUS.

Our authors observe that these conditions of the uterus have only very lately excited much attention. They have reason to believe that sometimes flexion of the uterus is congenital, at least, they have observed it in young females in whom the uterus had not undergone the changes that puberty, or copulation, or pregnancy entail. These cases, however, are very rare, and our authors think that the rapidity with which the uterus develops itself between the ages of twelve and fourteen, will explain the affection by the circumstance of one side being rather more tardy than another after pregnancy, or, on the other hand, if one side is reduced rather more rapidly than another, or if one of the parietes be more condensed than the other. In married women, the uterus inflamed and irritated may be elongated, softened, or retracted on one side, by the formation of an internal cicatrix, or even from irregular muscular contraction. At all events, married women present these incurvations much more frequently than the unmarried. What appears to confirm the idea that chronic inflammation is frequently the cause of these alterations is the circumstance that, on dissection, the uterus in such cases is often found of a deep red colour, or even blackish, or that there are present fibrous degenerations, or adhesions, or obstructions of the cavity of the cervix, &c. In a case related by Denman, that able author would seem to have looked on retention of urine at the period of delivery as the cause of retro-flexion, for the latter returned every time that the bladder was full and passed away on pushing the fundus of the uterus above the sacro-vertebral angle as soon as the bladder was emptied. Our authors have seen two cases of a similar description, but think that mere retention of urine would not produce the effect in question, if a peculiar condition of the uterus did not co-exist and co-operate with it. Besides retention of urine could not possibly produce ante-flexion.

Flexion operates more particularly towards the part where the neck and body of the uterus unite, and the curvature assumes various degrees of angularity. Sometimes it is such that the organ is actually folded on itself, and the two portions approach closely to each other. Commonly the curvature is rigid; rarely, if not soon after accouchement, there is mobility of the curved organ. And next for the means of determining the existence of this affection.

Before making an examination, the bladder and rectum should be emptied. The examination with the finger in vagina should be made in two different attitudes, upright and reclined, and the other hand should always be applied on the hypogastrium, both to ascertain whether the fundus of the uterus has its proper position, and also to depress the organ, and render both its cervix and body more accessible to the finger. It sometimes happens that the cervix is directed much backwards in retroflexion; but then the fundus is not inclined forwards, as in reversion; on the contrary, the fundus may be carried a little backwards also. A rounded or angular concavity is directed backwards, whilst the projection is directed forwards. If it is wished to obtain a very accurate examination, the uterus being firmly fixed by one hand applied on the hypogastrium, the extremity of the index finger of the other is to run over the curved portion of the organ, and so define its form. This is most easily done on the left side, if the right hand is employed in the examination. The same signs, in an inverse sense, characterise the anteflexion, but in this the orifice is found more constantly in the centre of the pelvis. In order to complete the diagnosis, we should attempt to move the uterus in several ways, to ascertain whether it be free or adherent. In this case, as in all that are doubtful, examination by the rectum may greatly assist. Whilst the fore-finger in the intestine touches the prominence formed by the uterus, or even its fundus, our authors have sometimes succeeded in passing into the vagina the thumb of the same hand, and in thus siezing the viscus in its length, and appreciating its condition with much precision. This is not difficult with women who have had many children, or abused sexual intercourse, nor with young women with habitual leucorrhœa.

Leucorrhœa frequently accompanies a flexion of the uterus, and amenorrhœa still more frequently. These complications, or effects of the uterine alterations, may make part of the characteristic signs of this kind of affection, to which other symptoms, hysterical, &c. may be added. The affection will be more or less serious, in proportion to the gravity or otherwise of these accidental symptoms. Sterility may also be a consequence; but our authors have seen three instances in which impregnation took place.

Pregnancy may, perhaps, produce a temporary or permanent cure; and, in order to prevent a relapse after parturition, the uterus should be carefully re-placed, its contraction accelerated by frictions on the hypogastrium, &c. In all cases of pregnancy or recent delivery, we should take care that the flow of urine be free, and the issue of the fæces sufficiently free and frequent. After parturition, we should endeavour to retain the uterus in a proper form by position; if there be anteflexion, the patient should lie upon her back; if retroflexion, she should recline upon her side, with an inclination as much forwards as possible. Our authors ask if it would be dangerous to introduce into the cavity of the uterus a wooden spatula, covered with lint and cerate, and maintain it there just long enough to allow the uterus to acquire some firmness. If the flexion is of some duration, and the uterus empty, this measure is of course improper. In such cases, our authors recommend commencing the treatment with local or general antiphlogistic measures, if any symptoms of inflammation or plethora exist. Mechanical reduction should be tried with the fingers, and, if it is effected, a piece of sponge, or an ivory or gum-elastic pessary, may be introduced into the bottom of the vagina, and applied in front of, or behind the os tincæ, as the case may require; or a ball-and-socket pessary may be used, with one side of the socket higher than the other, in order to raise the inclined part. Our authors doubt whether such instruments can be borne, or be useful, and recommend in such cases less specific means, such as stimulants over the cords, &c. which we need not dwell on.

Thirteen cases of morbid flexion of the uterus are related. We will briefly hint at one or two.

CASE.—*Anteflexion, apparently Congenital.* Miss Anna B. æt. 27, had begun to menstruate at 18, but this function had been very irregularly performed, and there was an abundant leucorrhœa. She complained of great weight in the perinæum, dragging at the groins and in the loins, increased on walking, standing, or straining at stool, and frequent attacks of hysteria. On examination, the os tincæ was found within an inch of the vulva, and on making more particular examination of the uterus, its fundus was found inclined upon the bladder; its volume was very small. Marriage was recommended.

CASE. *Anteflexion after Parturition.* Frances M., cook, æt. 24, who had been regular from the age of 16 years, married, and became pregnant a year after marriage. After some unusual exertions, she was seized with pains in the region of the kidneys, followed by violent uterine hemorrhage. She was at this time in the sixth month of pregnancy. Although the loss of blood was very great, this woman returned to a laborious employment in ten days. She remained feeble, pale, and complained of dragging sensations in the lumbar region and in the groins; constipation was extremely obstinate, and although she had been absent from her husband for ten months, she had not observed any menstrual secretion. The abdominal parietes were very lax, and whilst pressure was made on the hypogastrium with the left hand, the fore-finger of the right introduced into the vagina, allowed the form and situation of the uterus to be distinguished. The body was of very small size, and curved forwards; its fundus was in contact with the inferior border of the symphysis pubis, and the os tincæ rested almost in the centre of the vagina. We need not mention the treatment, for we are not told whether it was or was not efficacious.

The next case related is one of anteflexion, observed at the commencement of pregnancy; as the pregnancy advanced, the flexion sensibly and materially diminished.

The fourth case is one of retroflexion of the uterus in a girl of 18 years old, who died of hypertrophy of the heart. There was a tumour, of the size of a nut, on the fundus of the uterus; the tissue of the body of the viscus was red and softened round the tumour. The cavity of the cervix was obstructed by cretaceous matters, which had no doubt been an efficient cause of an amenorrhœa, under which the young woman had laboured. M. Dance has related a very similar case.

Case.—*Retroflexion after Delivery.* A female, in whom delivery was retarded by excessive distention of the bladder, presented the following condition of the parts. The fundus of the uterus, turned backwards, occupied and filled the pelvis, whilst the orifice was so raised behind the pubes, that, had it not been for the umbilical cord, it would have been impossible to have discovered it. Our author drew off more than four pints of urine, when the cervix descended into the pelvis in front of the body, and complete retroflexion was found to exist. In order to effect delivery, it was necessary to introduce the hand into the uterus, which was done with difficulty. As soon as it was effected, the fundus was raised, and made to pass over the sacro-vertebral angle, when a strong contraction effected at once the expulsion of the hand and the placenta, and maintained the organ in its proper position.

Case.—*Retroflexion after Abortion.* Madame C. a Creole, æt. 25, having never menstruated, married at the age of 14. In a few months she became pregnant, and had a long labour and painful delivery. Afterwards she went through her full time twice, without ill consequences. After this, she had in two years three abortions, between the second and fourth month. The last was followed by inflammation of the uterus. From this time, she always suffered from a painful sensation in the cavity of the pelvis, had painful menstruation at intervals of two and three months, and had symptoms of general derangement of the health. M. Dugés was invited by Dr. Rayer to examine the patient. He found the uterus affected with a slight retroflexion, and advised the patient to try the effect of a fresh pregnancy. She was at first much averse, the remedy being worse than the disease; but M. Dugés has since heard that she became enceinte, and miscarried at the third month.

We pass over the following chapter, dedicated to the subject of introversion of the uterus, as the English reader is already familiar with the affection, from the works of our own writers upon midwifery. We arrive, then, at the fourth section, which treats of distention of the uterus by foreign substances.

### DISTENTION OF THE UTERUS BY FOREIGN BODIES.

The cavity of the uterus may be filled, or the organ much distended, in several ways—by gas, by liquids, by solids. And, first, of the retention or inclusion of gas.

*Retention of Gas in the Uterus, or Physometry.* Our authors have only observed this phenomenon in obstetrical cases, where some remains of the membranes are retained in the uterus, or portions of the fœtus deprived of vitality, or putrid coagula. In such cases, the uterus may be more or less prominent in the hypogastrium and vagina, resonant on percussion, and forming a circumscribed tumour. Cases have been related, in which the air would appear to have been a morbid exhalation, and not the result of putrefaction. Such a case has recently been published by the Medico-Chirurgical Society of Bologna.

*Case.* A woman, aged 40, thought that she was pregnant; her menses were suppressed, the belly swollen, and the uterus, at the fifth month, at the level of the umbilicus. One day, whilst stooping, a great quantity of flatus escaped from the vulva, and the abdomen immediately subsided.

Women subject, as they sometimes are, to the escape of air from the vulva, usually experience it in stooping, or such motions of the body, in consequence, we may suppose, of the compression then exerted on the uterus. The expelled air is sometimes inodorous, and it is supposed that this is atmospheric air, accidentally introduced. Larger quantities must be artificially introduced, as in the case related by Frank, in which it was occasioned by the use of a liquid injection. Usually, however, as has been stated, it is the result of chemical decomposition, either of the putrefaction of the mucus of leucorrhœa, of the sanious discharge of a cancerous ulcer, or of clots from previous menorrhagia, or even of the menstrual secretion itself. Our authors relate, from Frank, some cases. We will give one.

*Case.* A woman had the menstrual secretion suppressed from exposure to cold. Pain and tumefaction of the uterus, which rose as high as the umbilicus, and was resonant on percussion, followed, and these symptoms were accompanied with fever in paroxysms. A finger was passed as high as the os tincæ, and a gust of fetid gas escaped. The belly immediately diminished in size, but it again swelled. A tube was introduced into the uterine cavity, a great quantity of gas issued from it—this was followed by the expulsion of coagula; and the patient was cured.

It is to the putrefaction of certain products of conception that we must attribute the expulsion of bladders filled with air, or flatulent moles, instances of which have been related.

The symptoms of this affection are sufficiently obvious. The prognosis must depend upon the nature of the affection producing the air. If merely the result of secretion, it is merely an inconvenience, and mechanical means will readily remedy it. Cleanliness, baths, lotions, injections, either of pure water or of chloride of lime, are all that, in ordinary cases, are necessary.

*Retention of Water, or Hydrometry.* This name is given to a collection of serous, sero-mucous, or albuminous fluid in the cavity of the uterus. This affection, like the preceding, is generally symptomatic. Our authors have seen it occur and disappear during the course of scirrhus of the uterus. They have also seen it follow a chronic metritis. Most commonly it has been found, on examination, mixed with pus and blood, and, in a few instances, the uterus was found simply extended and diminished in thickness. Most frequently, its tissue is studded with scirrhous indurations, ulcerations, hydatid or polypous tumours, and its orifice is sometimes obstructed by a tumour, sometimes merely closed by tumefaction of its borders. Thus, then, hydrometry is usually only a symptom. Our authors next allude to certain collections of fluid that take place during pregnancy, which may have a different seat in different cases, and sometimes occasion serious consequences.

1. It has been long remarked that women pretty far advanced in pregnancy, and especially about the middle of their time, may discharge a considerable quantity of water by the genital organs, without supervention of abortion. Puzos saw this take place four times during the same pregnancy. Some have thought that this quantity of water existed in the allantois; others between the laminæ of the decidua. Our authors incline to the former opinion.

2. The true waters of the amnion accumulate sometimes superabundantly in this membrane, and a state, really of disease, results, that may be termed *hydramnios*. Fifty pounds of water have been said to be so collected; but probably this is an exaggeration. Sometimes this affection has given rise to metritis, and traces of inflammation have been seen even in the placenta and membranes of the ovum. Perhaps the inflammation may have been rather the cause than the effect. In a case mentioned by Van Swieten, there were twins.

Our authors have seen a similar affection with a double pregnancy; dangerous peritonitis ensued, miscarriage occurred, and the twins were born dead. In a case mentioned by Doctor Duclos, the labour was premature, but the child was born alive. In other instances, the child has been born at the full time, but dropsical, or, as our authors have seen, encephalic.

True hydrometry has appeared to be sometimes occasioned by general causes, as a weak constitution, &c., but more frequently by some circumstance productive of local inflammation, as a blow upon the hypogastrium, &c., or by chronic inflammation. It has only been observed in young married women. It has appeared in various degrees, the uterus sometimes scarcely containing two pints of liquid, sometimes so distended as to counterfeit pregnancy. Sometimes even the quantity of fluid has been so considerable, that ascites was believed to be present, and eighty five pints of ichorous and oily-like matter *(Blanckard)* have been found in the womb. Vesalius even speaks of such a collection as one hundred and eighty pints of water.

The diagnosis may be difficult, as from the rarity of these cases they are not suspected. Fluctuation will distinguish this affection from scirrhous enlargements, &c. The obscurity of the fluctuation, and still more the distention of the uterus discoverable by the touch, will point out the difference between it and ascites, or ovarian dropsy. The absence of the motions of the fœtus, and of the pulsation of its heart, as detected by auscultation, will prevent it from being confounded with pregnancy; whilst the want of resonance on percussion will shew that it is not a collection of air.

The danger of the case must depend in a great degree on the cause of the collection of water. It will of course be less, when the obstruction at the neck of the uterus is incomplete, and allows, from time to time, the evacuation of the liquid. Fernel mentions a case where such an evacuation took place every month. Frank speaks of another in which pregnancy occurred twice in a woman subject to alternate retentions and evacuations of serous fluid. Such an evacuation may take place about the natural period of the termination of a supposed pregnancy, and Mauriceau, Nanshe, and others have seen this not followed by any relapse.

The treatment must be guided by and directed against the disease of which this is the consequence. In itself, and considered idiopathically, we may be compelled to attend particularly to it, on account of the inconvenience it produces. Monro observes, that a mechanical succussion, that of vomiting, &c. may occasionally produce the discharge of the fluid. At other times the finger, or a blunt stilet may be introduced by the vagina, into the os tincæ, and the orifice be opened; or a tumour falling down upon the orifice might be pushed up in the same manner. In some severe cases the inefficiency of these means may render puncturing with a trocar necessary. This has been done with success above the pubes, and 53 pints of thick, black, and bloody fluid have been thus drawn off in a female of 53 years of age: ten months after this there was not the least appearance of a relapse. A similar puncture was made with advantage for a hydramnios, which threatened the life of the woman. Undoubtedly it would be better to puncture the membranes through the os uteri, if it were possible; a long trocar, of a probe curve, would be the best means of doing so. Cruveilhier mentions a case in which this operation proved fatal. Our authors relate one case, which is sufficiently interesting to be noticed here.

*Case.* A young lady, of strong constitution, had complained for a long time of violent pain in the right iliac region, and of the sensation of a ball, accompanied with burning heat, passing from the uterus to the œsophagus. On the 1st of October, 1829, after violent pains in the hypogastric region, she gave issue, at several times, at intervals of five minutes, to a pint of colourless, inodorous, liquid, staining the linen like the serum of milk.

On examination the uterus was found lower, the edges of the orifice thicker, and the orifice itself more widely opened than natural. There was a point which when touched occasioned pain, nor could anything particular be distinguished in the hypogastrium.

*Calculi of the Uterus.* These, of course, are very rare, so much so that M. Roux attributes their formation merely to the ossification of fibrous tumours, which are known to be very common. A calculus of the uterus which was analyzed some years ago was found to consist of a large proportion of animal matter, united with salts of potassa, soda, and lime. Another, which M. Amussat examined, was composed of phosphate of lime and gelatine. M. Louis has collected and published, in the Memoirs of the French

Academy of Surgery, the greater number of the cases that have been recorded.

The symptoms appear to have been—feeling of weight, difficulty in walking, pruritus of the vulva and at the top of the thighs in one case, whilst other women, in whom the stone has been smaller, have not suffered much inconvenience, and others, again, have had very serious consequences, such as ulcerations, &c. and have even died in consequence. Symptoms then such as these—tumefaction of the abdomen, colicky pains, sensations of weight, difficulties in the emission of the urine and the fæces, induration of the uterus when felt from the vagina, may lead to the suspicion of the existence of uterine calculi, but their presence can only be rendered certain by feeling the stone itself either with the finger or an instrument.

Louis cites three or four cases in which the stone issued spontaneously, or was extracted from the vagina. He mentions a case in which a stone was extracted by incision of the uterus, but he does not relate any details. He recommends the stone to be sought for and extracted with forceps, after having suitably enlarged the os uteri with the knife. In a case related in the Revue Medicale, and purporting to be taken from a Turin Journal, the orifice was sufficiently open without any further enlargement.

Passing over a not uninteresting chapter on Retention of the Menstrual Secretion, we arrive at the sixth chapter of the section, dedicated to the consideration of Moles.

*Moles.* Our authors limit these to three kinds:—1, the false germ: 2, the fleshy mole: 3, the hydatid mole. In this point of view, moles are always a product of conception, and cannot exist independent of fecundation.

I. Our authors contend that if the causes of abortion are often exclusively maternal, they may also be found in the state of the embryon itself. Thus it occasionally happens, that on opening the membranes corresponding exactly to a human ovum at an early period, only water is discovered, or perhaps some filaments adhering to the membranes, floating in the liquid, and probably the remains of the umbilical cord. It is this that should be called more particularly and properly a false germ.

A false germ seldom remains in the uterus for more than three or four months, and it is impossible to distinguish it from a pregnancy of the same period, whilst its expulsion does not differ from an abortion, properly so called. It is commonly expelled entire. If it breaks previously to expulsion, the waters escape, and it becomes impossible to say at an early period whether or not an embryon detached with its envelopes has been expelled and lost amongst the coagula which come away before the membranes. These membranes are always composed of the amnion, chorion, and decidua. The placenta may be more or less circumscribed or developed; most commonly the filaments which constitute it are still universally spread over the surface of the chorion, and hidden in the decidua; the latter appears in consequence very thick, the thickness, however, being often accompanied with increased consistence owing to the fibrinous coagula infiltrated into its tissue. This alteration is distinguished with more difficulty when the false germ has been preserved for a length of time in alcohol, and it is then very easily confounded with the fleshy mole.

II. The fleshy mole differs from the false germ only in its longer sojourn in the uterus, and in the more complete changes which it undergoes. Our authors think it may easily be conceived, that if the false germ, or the ovum deprived of the embryo, remain attached to the uterus, and absorb and appropriate to itself the blood destined to the fœtus, it will not only acquire volume but compactness. Our authors describe two varieties of fleshy mole.

1. Sometimes it is hollow in the centre, and contains water, but the cavity is always very small in proportion to the thickness of the parietes. These are unequally dense, red, fungous, somewhat like the tissue of the placenta. The mass is more or less regularly rounded, or ovoid, sometimes lobulated, &c. and varies in size from that of a large egg to that of a child's head.

2. Sometimes more irregular and often more voluminous, this mass presents no longer a central cavity, whether it be obliterated by absorption of the liquid, or whether that and the embryon with it flowed out through some aperture. Very large productions of this sort have been recorded, but the greater number have not exceeded the two fists in dimension. The tissues of which they are composed are various:—in one part we see a filamentous tissue as spongy as that of the placenta; in another a compact and parenchymatous substance; hydatid vesicles are scattered amongst it, fibrinous clots intermixed with it, and sometimes even remains of the fœtus, such as bones, &c. are incorporated with it, or adherent to it.

This degeneration of the fœtal envelopes may be met with in a case of twins, as well as in one of simple pregnancy, and one only of the products may be affected, the other pursuing its normal development to the full time, when the mole is expelled with the secundines of the healthy infant, or some days after it. Sometimes this complication has not allowed gestation to be completed, and a premature labour has been the necessary consequence. More rarely the mole is expelled prematurely, at seven months for instance, and pregnancy continues and terminates in the regular manner. Some examples of two moles existing in the same uterus have been related. These may become united. The epichorion is usually common to the two products of a twin conception, and this may serve to unite them. It has been seen encrusted with calcareous salts, and forming on a mole an envelope of osseous consistence.

The diagnosis of a fleshy mole is very often difficult, especially in the first months, in fact we may say that its symptoms at that period do not differ from those of a painful pregnancy. The symptoms may be described as being—a feeling of weight and distress in the region of the uterus, and small losses, more or less frequent, of serous blood. These are sufficiently uncertain. At a later period we may observe that the belly is larger, and projects more in the hypogastrium than the presumed period of pregnancy would warrant; this prominence, too, appears more resisting, and the uterus, as felt from the vagina, is more compact, more unequal, more weighty than it should be. The feeling of the weight to the patient herself increases, and the womb appears to fall to the side to which she inclines. If the period is still later advanced, we seek in vain for the motions of the child, and the pulsation of its heart.

One of our authors, M. Dugès, lately saw a case which proved how difficult it is to decide upon these points. A child was born at five months, when the placenta, which had been fixed at the orifice of the neck had been gradually detached. Continual evacuations ensued; they were chiefly serous, but always coloured with blood, and always debilitating. All the signs enumerated above had existed, and no doubt could have been entertained of the existence of a mole, if the patient had not been confident that she felt the motions of the child.

Thus, then, we are often uninformed of the existence of a mole till it is expelled. The expulsion is generally slow, painful, preceded, like an abortion, by repeated hæmorrhages; sometimes, however, it is easy and rapid. The unfavourable mode of expulsion is probably owing to the adhesions which the fleshy mass has frequently contracted with the uterus, in consequence of its partial separation, and also of the condition of the uterus itself, a condition approaching to one of disease, if not actually such. Usually the fleshy mole is expelled from the uterus some months later than the false germ, shewing, or seeming to shew, that it is only an advanced degree of the latter; and that the same product, expelled early, is a false germ—retained later, is a fleshy mole. Those of great dimensions had exceeded the natural term of gestation; but the greater number were expelled between the third and sixth month, and commonly nearer the former than the latter.

The hæmorrhage which precedes or accompanies the expulsion may be serious; Delamotte has seen it fatal. Usually, however, the result is not unfortunate, and we need not even despair of subsequent pregnancies and natural deliveries, although some women would seem to present an inherent disposition to the affection.

Treatment for this, as for the false germ, must be almost entirely palliative. Sometimes plugging may be necessary for extreme hæmorrhage; sometimes the ergot of rye, stimulating layements, fumigations, hip-baths, injections, will be necessary to quicken the contractions of the uterus, or favour the separation of the mole and the dilatation of the cervix. For the latter object, our authors mention that the ointment of the extract of belladonna may be useful; the fingers, even the hand itself, or the false germ forceps of Levret, or the blunt hook of Fabricius Hildanus, may mechanically expedite a slow, difficult, or incomplete expulsion.

III. Our authors next advert to the vesicular or hydatid mole. This is the product of another sort of degeneration in the envelopes of a fœtus. Here, however, the degeneration appears to be the cause of the loss of the embryo, and not, as in the preceding cases, the effect. To prove this, our authors enter on the following considerations. When, in the first months of gestation, we examine carefully the filaments which spring from the chorion and plunge into the decidua, to constitute the placenta, we find them interwoven, a circumstance which was mistaken by some ancient writers, sometimes for distended lymphatic vessels, sometimes for glands. From this circumstance, Vallisnieri was of opinion, that uterine hydatids are varicose enlargements of those lymphatic vessels. Our authors observe, that the enlargements presented by the villosities of the chorion, and the filaments of the placenta, are purely of a cellular and spongy nature. In this structure, our authors appear to place the hydatids, and to agree, in some measure, with Vallisnieri in opinion.

Our authors describe three varieties of mole; the vesicular embryon mole (mole embryonnée)—the hollow vesicular mole—and, finally, the vesicular mole en masse. Those of our readers who are curious on this head we must refer to the work itself, or to the translation, which is now, we understand, in process of publication.

Our authors remark that the symptoms, duration, and termination of the vesicular mole, differ little from those of the fleshy mole. The uterus, however, is in general less heavy and less hard, although fluctuation is not more distinct; the duration of this false pregnancy is longer than in the instance of the fleshy mole, and ranges from three to ten months; and, finally, these false hydatids are more frequently expelled, not at once, but at several intervals. The symptoms, again, are more serious, and death more frequently results.

Four cases are related. In the first, the hydatid mole was expelled at the end of twelve months and a half after impregnation—in the second, it was expelled at the fourth month—in the third, at the eighth—and, in the fourth, at the seventh month. We will confine ourselves to a narration of the first case.

*Case.* Madame D. æt. 34, of lymphatic temperament, experienced all the symptoms of pregnancy, and the belly acquired a moderate size for five or six months, raising every expectation of a fortunate accouchement. At the ninth month there were no pains, and she consulted several medical men. One accoucheur said that she was enceinte, but remarked that the pregnancy was only of some months. MM. Bourgeois and Marjolin were of the same opinion. Six months elapsed, and nothing fresh occurred. Fifteen days after this, that is, twelve months and a half from the first suppression of the menstrual secretion, the patient was seized during the night with violent pains, followed by violent loss of blood. M. Lambert was summoned, and extracted a mole, which was lacerated during the extraction. It contained in its cavity, which was recognized as that of the amnios doubled on the chorion, a liquid, the quantity of which was estimated at eight ounces. The membranes lining this cavity were surrounded by a considerable quantity of very transparent hydatids, varying in size, from that of an almond to the smallest grain of sand. Several of these hydatids projected into the cavity, and a group appeared to have been the origin or the remains of an umbilical cord. Externally, this mass was lobulated, granular, and like the external surface of a placenta at full term.

Neither during the gestation of this mole, nor afterwards, was there any distention of the mammæ, nor was there any loss of blood during the gestation. The lochia flowed for several days afterwards.

The fifth and last section of the volume before us is devoted to the consideration of excrescences and degenerations. It comprises three chapters; the first on Excrescences and Degenerations in general—the second on Fibrous Tumours having no Pedicle—the third on Fibrous Tumours having Pedicles. We fear that we can only notice the first chapter. The other portions of this and the succeeding volumes, as they appear, will be fully considered, and, if necessary, analysed in this Journal. A translation of the work is, we believe, appearing, and we do not doubt that either that translation, or the original, will, after the attention we have drawn to it and the specimens we have given of it, be considered a valuable addition to the library of the obstetrical physician, or the general practitioner.

### Degenerations and Excrescences.

These are divided by our authors into seven varieties—vascular, cellular, fibrous, osseous, tubercular, steatomatous, cancerous.

*Vascular Degeneration.* These are what Levret denominated *vivaces*, and which he very properly discriminated from polypi. From their rapid reproduction, probable origin, and from the facility with which they bleed, they appear to be formed essentially of new capillary vessels. Our knowledge with regard to them is still imperfect. Are the ulcerations which give rise to them cancerous in the first instance, or do they subsequently become so? Our authors merely mention two cases related by Hervez de Chégoin.

*Case.* A woman, æt. 50, had been subject for a year to frequent hæmorrhages from the uterus. On examination, M. Hervez found implanted on a prominent point of the os tincæ, at the posterior lip, a fleshy growth, granular, three inches in length. It was so soft, that each examination detached portions of it. A ligature placed upon its point of origin cut it through abruptly, and a violent hæmorrhage followed, but was checked by plugging. For eight months, there was no appearance of return of the disease; but the point of origin of the tumour remained a little elevated. Six months later there was a return of a similar fungous growth, of the volume of an almond, and, at the same period, different tumours were developed around the uterus, with pyrexia, and all the symptoms of cancerous contamination of the constitution.

*Case.* A woman, æt. 48, and equally subject to hæmorrhages, accompanied with violent pains in the uterus, presented, when examined, vegetations springing from within the os uteri, which was partially open. Several fragments of these, of a reddish-brown colour, were expelled at different times. Some similar excrescences shewed themselves on the os tincæ, and a decided cancerous diathesis was established, and speedily killed the patient.

Our authors observe that those excrescences which have frequently a large base, an unequal irregular surface, granular, red, bleeding, and without external membrane, are but a form of cancer.

There is another form of vascular production or excrescence, which our authors rather refer to fungus hæmatodes. They have, on several occasions, found women subject to frequent hæmorrhages from the vagina present, on examination, no other lesion than a painful swelling of the os tincæ, a softening, sometimes with superficial ulcerations, a violet colour, and always a great readiness to bleed on the slightest contact. This state has sometimes changed into a true degeneration of the cervix uteri, which, becoming a violet or brown colour, and bleeding readily, has terminated by tumefaction and extreme softening, and, finally, by ulceration and total destruction. These cases, which our authors will notice more at length hereafter, appear to them to answer to the character of fungus hæmatodes.

*Cellular Excrescence.* This resembles the polypus of the nose, but it is not common. In order to illustrate the affection, our authors again borrow from M. Hervez de Chégoin.

*Case.* A woman, æt. 50, had been subject for seven months to continual losses of blood. The uterine orifice, partially open, allowed a smooth, rounded, not painful, projection to be felt. It was attempted to place a ligature upon it, but it slipped from out the knot; to seize it with a hook, but this tore its way out. It was finally laid hold of with the forceps, and tied high up; but the ligature cut the pedicle, and brought away a polypus of the form of a fig, of a reddish-brown colour, and an inch and a half in length. Pressure forced blood out of it, and reduced its volume considerably.

In other cases, these excrescences have arisen from the os tincæ. One which had not been suspected during life was of the form and size of an almond, soft, reddish-brown, streaked with very small vessels, and easily detached from the surface, to which it hung by a very fine pedicle. The same observer has remarked in the dead body three such excrescences, two fixed at the fundus—the other at the cervix. The exterior of these little tumors were continuous with the tissue of the uterus, and internally with its substance. Our authors have seen many instances of small excrescences, but they rather partook of the fibrous or fleshy character, and, on the whole, they are are inclined to think these cellular excrescences rare.

The treatment does not differ much from that of fibrous polypi, of which they intend to speak anon. They think however, that they may be torn away, as nasal polypi are.

*Fleshy and Fibrous Degenerations.* It is so impossible to draw the line between these and the fibrous tumours of the uterus, which have been termed polypi and fleshy tubercles, that our authors will consider them all under one head.

*Cartilaginous and Osseous Degenerations.* Fibrous and tubercular tumours and excrescences may gradually assume so much induration, as to acquire the consistence of cartilage, or even of bone. The whole uterus may be thus converted into an osseous structure. Louis relates three cases of ossification of the whole uterus. A woman, who died, at the age of 60, of consumption, and carried for twenty years a hard tumour in the hypogastrium, presented, when examined by the surgeon, Mayer, an osseous uterus, the parietes of which were four lines in thickness, and its anterior was filled with pus. Hooper mentions his having seen an uterus of ordinary size, irregular on its surface, composed of an osseous mass, and covered by a layer of fibres, and in some points by peritoneum. On chemical analysis, it was found to consist of carbonate and phosphate of lime, with some animal matters.

Partial ossifications have been alluded to by many authors, but in none was the tumour of unmixed character. Hooper appears to have seen insulated and well-circumscribed cartilaginous tumours in the substance of the uterus. Our authors inquire, very properly, whether these were not fibrous tumours of rather more firm consistence than usual; for the consistence of fibrous tumours is, it is well known, variable.

The prognosis is not so light as would à priori be imagined, for many patients die. It is true that they seldom have any symptoms that rapidly prove fatal, and patients seldom sink in early life. Art can do little or nothing.

*Tubercular Degeneration.* This is one of those which is rarely seen separately in the uterus, but most commonly mixed with others. At the present day the term is limited to that alteration or production in which the substance of an organ is infiltrated with, covered by, or merely has deposited in it a mass of concrete, white or yellowish, rather albuminous matter, varying in consistence from that of a chesnut to that of a puriform bouillie. In some cases Madame Boivin believes that the tubercle arises, as Dr. Baron believes, in a primitive vesicle, but in other instances our authors seem to think that it consists of a simple depôt of a sort of concrete pus in the texture of the part affected, in some natural interspaces of an organ, or sometimes on its free surface. Such are the greater number of the tuberculous productions in the uterus; they are rarely seen in its tissue, usually on its external or internal surface.

Tubercle on the *external* surface is sometimes insulated, prominent, rounded, encysted under the peritoneum, but adhering by a large base to the tissue of the uterus. Tubercles are frequently seen on dissection, in conjunction with more serious lesions. It is very usual to see tubercles external to the uterus after repeated attacks of peritonitis which has become chronic, or after inflammations of the uterus to which prolonged inflammation of the hypogastric and pelvic peritoneum has been joined. They are found in the midst of adhesions contracted by the uterus with its appendages and with contiguous serous surfaces. We see even under the peritoneum and in the cellular tissue uniting it to the uterus, masses of greater or less volume, but more commonly of miliary character, sometimes separate, sometimes numerous and confluent, of greyish or white tuberculous deposite, mixed even with black colouring matter.

In the *interior* of the uterus, in certain cases of amenorrhœa apparently caused by chronic inflammation of the organ, we see deposited a concrete, adherent, thick layer of white or whitish matter, similar to that of tubercles. In most of these cases of tuberculous degeneration there is an evident scrofulous diathesis. In some cases tubercles have also been found in many other organs, both near to and remote from the uterus. Two cases of this kind have been published by Dr. Kenard. The woman had borne many children; the organs affected were the lungs, the peritoneum, and the uterus. In one of the cases there were also ulcerations of the vagina, and tuberculous matter deposited in the fallopian tubes, one of which was obstructed. A case is also related by our authors.

*Case.* Mademoiselle V. C., æt. 16½, had been very imperfectly nourished in early infancy, and had long suffered in consequence. Towards the age of puberty she had profuse leucorrhœa, but till the age of fifteen and a half menstruation was regular, when it ceased, and the leucorrhœa became still more profuse. About the same time she became affected with habitual constipation and swelling of the abdomen, accompanied with pain in its right side. Diarrhœa, vomiting of greenish matters, abundant metrorrhagia followed; she died in a state of marasmus.

On dissection there were found tubercles in the lungs and in the peritoneum, with conversion of the omentum into an adherent granular mass. The ascending colon was dilated, and its parietes almost cartilaginous. The left ovary was enlarged and tuberculous; the opposite ovarian tube studded with three encysted tubercles; the right ovary filled with a mucous substance of blueish black colour. The uterus was red externally, and coated internally with a whitish and granular matter a line in thickness.

*Steatomatous Tumours.* True steatomatous tumours are very rarely seen in the uterus, though many examples are on record of other alterations and affections having been designated steatomatous. Thus extra-uterine pregnancy, and development of the embryo in the substance of the uterus itself have been so confounded with steatoma. An instance of these ambiguous affections has been related by M. Delpech. In it hairs, bones, and fat, issued or were extracted by the urethra, owing to a communication which had taken place between the cyst and the bladder. Fabricius Hildanus mentions the case of a widow in whom the uterus was partly filled with a yellowish ichor, in part with fat and hairs.

The conclusion of the chapter contains a short notice on the subject of cancer of the uterus, which will be treated, hereafter, more in detail. Our authors observe that they admit four varieties of uterine cancer:—scirrhous cancer—ulcerated cancer—fungous cancer—and, cancer hæmatodes.

Here we must conclude the present article. Any lengthened expatiation on the merits or demerits of the volume before us would be out of place. We have noticed it so fully that our readers may ascertain whether it suit their tastes or not, and to our readers we leave the decision. But we may be permitted to observe, that the exact character and precision of the work are calculated, in our opinion, to render it a very useful addition to obstetrical literature in this country, and to add to the certainty of obstetrical practice. We are in the habit of attending to the effects of medicines, to symptoms, to circumstances more immediately and strikingly practical, and too frequently neglect those exact investigations into the varieties of disease, and into the differences of organic lesions, which, though they may appear to many minute and unnecessary, are on the whole useful, and give, when every subtraction of refinements and over-drawn distinctions is allowed, a demonstrative and definite character to the science. It is on this account that we feel disposed to lay before the profession the analyses of such works as the present, because we believe that they are calculated to correct much of that slovenly grasping at what appears immediately useful, which characterises too frequently English practitioners.

